# Impact of Liver Fibrosis on Survival of Patients with Intrahepatic Cholangiocarcinoma Receiving Gemcitabine-Based Chemotherapy

**DOI:** 10.3390/jcm11072057

**Published:** 2022-04-06

**Authors:** Maximilian N. Kinzler, Christina Klasen, Falko Schulze, Eva Herrmann, Andreas A. Schnitzbauer, Jörg Trojan, Stefan Zeuzem, Peter J. Wild, Dirk Walter

**Affiliations:** 1 Department of Internal Medicine I, University Hospital Frankfurt, Goethe University Frankfurt, 60590 Frankfurt am Main, Germany; maximilian.kinzler@kgu.de (M.N.K.); christina.klasen@t-online.de (C.K.); joerg.trojan@kgu.de (J.T.); stefan.zeuzem@kgu.de (S.Z.); 2 Dr. Senckenberg Institute of Pathology, University Hospital Frankfurt, Goethe University Frankfurt, 60590 Frankfurt am Main, Germany; falko.schulze@kgu.de (F.S.); peter.wild@kgu.de (P.J.W.); 3 Institute of Biostatistics and Mathematical Modelling, Goethe University Frankfurt, 60590 Frankfurt am Main, Germany; herrmann@med.uni-frankfurt.de; 4 Department of General, Visceral, Transplant and Thoracic Surgery, University Hospital Frankfurt, Goethe University Frankfurt, 60590 Frankfurt am Main, Germany; andreas.schnitzbauer@kgu.de; 5 Wildlab, University Hospital Frankfurt MVZ GmbH, 60590 Frankfurt am Main, Germany

**Keywords:** liver fibrosis, chemotherapy, overall survival, intrahepatic cholangiocarcinoma

## Abstract

Intrahepatic cholangiocarcinoma (iCCA) is the most frequent subtype of cholangiocarcinoma (CCA), and the incidence has globally increased in recent years. In contrast to surgically treated iCCA, data on the impact of fibrosis on survival in patients undergoing palliative chemotherapy are missing. We retrospectively analyzed the cases of 70 patients diagnosed with iCCA between 2007 and 2020 in our tertiary hospital. Histopathological assessment of fibrosis was performed by an expert hepatobiliary pathologist. Additionally, the fibrosis-4 score (FIB-4) was calculated as a non-invasive surrogate marker for liver fibrosis. For overall survival (OS) and progression-free survival (PFS), Kaplan–Meier curves and Cox-regression analyses were performed. Subgroup analyses revealed a median OS of 21 months (95% CI = 16.7–25.2 months) and 16 months (95% CI = 7.6–24.4 months) for low and high fibrosis, respectively (*p =* 0.152). In non-cirrhotic patients, the median OS was 21.8 months (95% CI = 17.1–26.4 months), compared with 9.5 months (95% CI = 4.6–14.3 months) in cirrhotic patients (*p =* 0.007). In conclusion, patients with iCCA and cirrhosis receiving palliative chemotherapy have decreased OS rates, while fibrosis has no significant impact on OS or PFS. These patients should not be prevented from state-of-the-art first-line chemotherapy.

## 1. Introduction

Cholangiocarcinoma (CCA) represents a heterogenous group of highly malignant cancers with a poor prognosis emerging at any point of the biliary tract. Despite the relatively rare occurrence, the incidence of CCA has increased globally and it is still the second most common primary hepatic cancer after hepatocellular carcinoma [1,2]. Intrahepatic cholangiocarcinoma (iCCA) is the most frequent subtype of CCA in Germany, while perihilar and distal CCA are more common in the United States [3,4].

Depending on the tumor stage, various therapeutic options can be distinguished, i.e., surgical resection, systemic chemotherapy and regional therapy, as well as recently emerged targeted therapies [5,6]. Since the majority of CCA is unresectable at the time of diagnosis, neoadjuvant chemotherapy, locoregional therapies including transarterial chemoembolization (TACE) or selective internal radiation therapy (SIRT) as a conversion to surgery as well as optimal palliative chemotherapy are crucial in this scenario [6]. Since 2010, the combination of gemcitabine and cisplatin is the standard of care for palliative first-line treatment [7]. A potential new first-line regimen was recently presented with the phase III clinical trial TOPAZ-1 [8]. To date, there is only one evidence-based second-line therapy [9].

To date, several risk factors for iCCA have been described such as viral hepatitis, liver fluke infection, hepatolithiasis, diabetes, primary sclerosing cholangitis or liver cirrhosis [10,11,12]. Nonetheless, the vast majority of CCA occurs sporadically, without the presence of an underlying disease [10,13]. Merely a few prognostic factors have been investigated in recent years, including tumor size, surgery, pathological grade or Union for International Cancer Control (UICC) stage [14]. The role of liver fibrosis as a prognostic marker for survival is still controversially discussed [15,16,17,18]. Moreover, data of the potential influence of fibrosis on the outcome of patients receiving chemotherapy are currently missing. Thereby, a potential impact of fibrosis on the clinical course of patients in the palliative setting remains unclear.

Against this background, we aimed to retrospectively assess liver fibrosis in patients with iCCA receiving gemcitabine-based chemotherapy and to investigate its potential impact on the clinical outcome.

## 2. Materials and Methods

### 2.1. Database and Study Population

**Figure 1:**
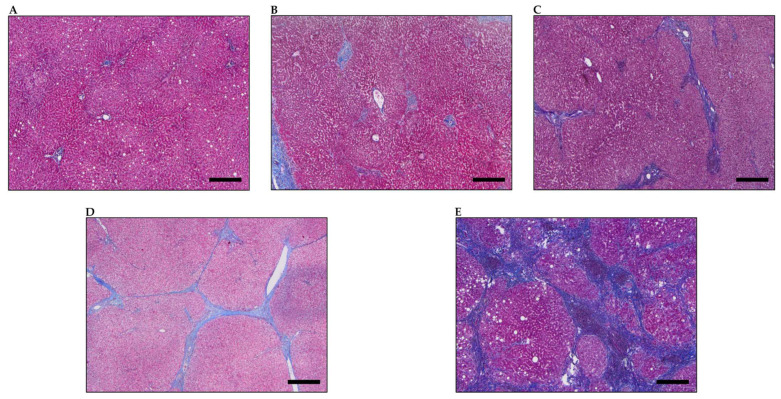
Representative images of Desmet score 0–4. (**A**–**E**) Representative liver histopathology of fibrosis score 0 (**A**), 1 (**B**), 2 (**C**), 3 (**D**) and 4 (**E**) referring to Desmet et al. [19] in Masson’s trichrome stain. Original magnification x4. Scale bars = 400 µm.

All patients treated with intrahepatic cholangiocarcinoma undergoing palliative chemotherapy at Frankfurt University Hospital between December 2007 and December 2020 were retrospectively analyzed. In total, we screened 50 patients with recurrence after initial surgical resection, as well as 162 patients being unresectable at time of first diagnosis. Next, we excluded patients who died within the first month of onset of palliative disease due to the impossibility to administer chemotherapy, along with patients lost to follow-up or in best supportive care. Of those remaining, patients without gemcitabine-based chemotherapy, external histology or patients lacking liver parenchyma in the tumor biopsies were not further investigated (Supplementary Figure S1). Patients with available formalin-fixed paraffin-embedded tissue samples were reviewed if tumor-adjacent liver tissue was available. All samples were retrieved from the archive of the Dr Senckenberg Institute of Pathology, University Hospital Frankfurt. Liver fibrosis score was assessed by an expert gastrointestinal pathologist, while low fibrosis was defined as score 0–2 and high fibrosis as score 3–4, referring to Desmet et al. [19]. Representative images of liver fibrosis Desmet score 0–4 are shown in Figure 1. Child–Pugh score was assessed according to common criteria [20]. Clinical data (date of birth, gender, tumor stage, tumor size, chemotherapy, laboratory parameters and follow-up) were collected from electronic medical records. Only patients who underwent gemcitabine-based chemotherapy as a first-line regimen were included. iCCA were staged according to the 8th edition of the classification of the UICC. The study protocol was approved by the local ethics committee of the University of Frankfurt (Approval No. SGI-1-2020).

### 2.2. Statistical Analysis

We compared the baseline clinicopathological characteristics between patients with low and high fibrosis scores. Categorial variables are presented as frequencies and percentages; continuous variables are shown as means with standard deviations. Categorial and continuous variables were compared using the Student’s t-test and chi-square test, respectively. Overall survival (OS) was defined as the time of onset of the palliative disease until death or date of last follow-up. Progression-free survival (PFS) was defined as the onset of palliative disease to its first progression or the first timepoint of the discontinuation of chemotherapy. In the case of initial surgical resection in a curative intention, palliative disease was defined as the onset of recurrence. Patients with exploratory laparotomy were defined as irresectable. Date of last follow-up was treated as a censored observation.

Survival was compared using the log-rank test. The Kaplan–Meier curves for survival were derived to visualize the comparison between low and high fibrosis scores as well as non-cirrhotic vs. cirrhotic patients.

Cox regression analysis was performed to assess the risk factors influencing patient survival. We preliminarily used univariate Cox regression analysis to screen our variables. We then included the variables with *p* < 0.05 into the multivariate Cox regression analysis. The adjusted common odds ratios are reported with 95% CIs to indicate statistical precision. The significance level was set to *p* < 0.05. All data were analyzed with SPSS 27 (IBM; Armonk, BY, USA) statistical software.

## 3. Results

### 3.1. Patients and Clinical Characteristics

In total, 70 patients with iCCA undergoing gemcitabine-based chemotherapy were analyzed. In line with widely known data, 67.1% (n = 47) of the patients were unresectable, while 32.9% (23) initially underwent surgical resection with a curative intention [6,21]. Of the identified patients, 70% (49) were assigned to the low-fibrosis group, while 30% (21) were in the high-fibrosis group. Reaffirming our pathological classification, the mean fibrosis-4 index (FIB-4) was 1.9 and 4 in the low- and high-fibrosis group, respectively [22]. In addition, 18.6% (13) were pathologically assessed as liver cirrhosis (Desmet 4). Shedding light on the hepatic function, we assessed the Child–Pugh score in our cohort. However, the hepatic function did not differ significantly between both groups. To be more precise, 76.9% (10) and 23.1% (3) of cirrhotic patients were diagnosed as Child–Pugh A and B, respectively. Cirrhosis already clinically occurred before the histopathological assessment in 76.9% of the affected patients. Overall, 81.6% (40) of the low- and 85.7% (18) of the high-fibrosis groups were treated with first-line chemotherapy gemcitabine/cisplatin, while the remaining patients received other gemcitabine-based chemotherapy regimens [7]. Baseline clinicopathological characteristics are shown in Table 1. Second- and third-line chemotherapy regimens are summarized in Supplementary Table S1.

### 3.2. Impact of Liver Fibrosis

**Figure 2:**
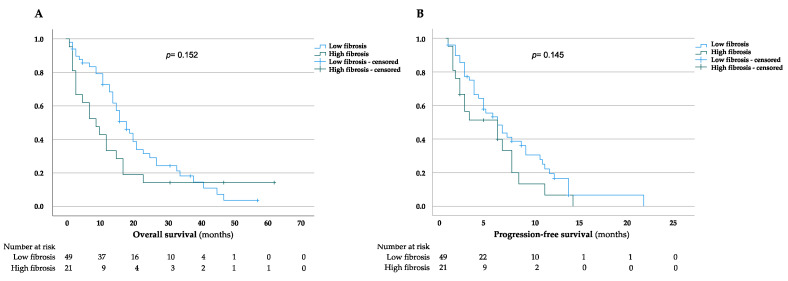
Kaplan–Meier curves for overall and progression-free survival in low and high liver fibrosis groups. (**A**,**B**) Overall survival (**A**) and progression-free survival (**B**) assessed for low and high liver fibrosis scores. Date of last follow-up was treated as a censored observation.

The median OS for patients with low and high fibrosis scores was 21 (95% CI = 16.7–25.2 months) and 16 months (95% CI = 7.6–24.4 months), respectively (Figure 2A). The three-, twelve-, and twenty-four-month OS rates were 89.7, 72.7, and 31.5% in patients with a low fibrosis score and 66.7, 33.3 and 14.3% in patients with a high fibrosis score, respectively. However, the improved survival rates in the low-fibrosis group were not statistically significant (*p =* 0.152). We further evaluated the impact of liver fibrosis on PFS in our study cohort. The median PFS for patients with low and high fibrosis scores was 6.9 months (95% CI = 5.3–8.6 months) and 4.7 months (95% CI = 2.9–6.5 months), respectively, while the three-, six-, and twelve-month PFS rates were 66.4, 43.6 and 16.7% in patients with a low fibrosis score, compared with 51.3, 33.2 and 6.6% in patients with a high fibrosis score (Figure 2B). In line with the OS analysis, we could show that a high liver fibrosis score is, indeed, associated with a shorter PFS in patients with iCCA receiving gemcitabine-based chemotherapy, although these results did not reach statistical significance (*p =* 0.145). Furthermore, subgroup analysis revealed that OS and PFS did not differ significantly for a low and high fibrosis score in patients initially undergoing surgical resection or being unresectable when diagnosed, respectively (Supplementary Figures S2 and S3).

### 3.3. Impact of Liver Cirrhosis

**Figure 3:**
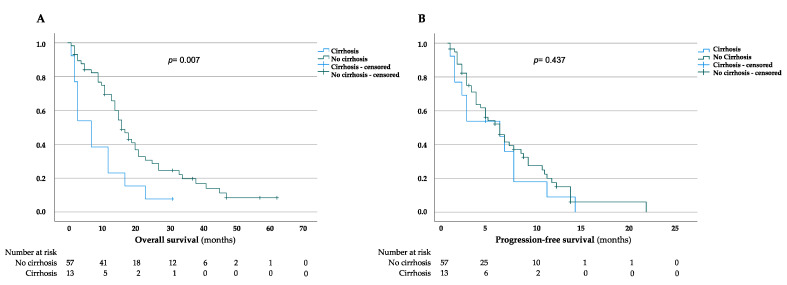
Kaplan–Meier curves for overall and progression-free survival in cirrhotic and non-cirrhotic patients. (**A**,**B**) Overall survival (**A**) and progression-free survival (**B**) in the presence and absence of liver cirrhosis. Date of last follow-up was treated as a censored observation.

The median OS for patients without liver cirrhosis was 21.8 months (95% CI = 17.1–26.4 months), in comparison to 9.5 months (95% CI = 4.6–14.3 months) for patients diagnosed with liver cirrhosis, thus showing a significant difference between both groups (*p =* 0.007) (Figure 3A). This was in line with the three-, twelve-, and twenty-four-month OS rates, which were 89.4, 69.4, and 30.7% in patients with liver fibrosis and 53.8, 23.1 and 7.7% in cirrhotic patients, respectively. To further strengthen the role of cirrhosis on the outcome of iCCA patients, we evaluated the PFS in both cirrhotic and non-cirrhotic patients (Figure 3B). However, our data revealed that the median PFS for patients without cirrhosis was 6.6 months (95% CI = 5.1–8.1 months) in contrast to 5 months (95% CI = 2.6–7.4 months) for cirrhotic patients (*p =* 0.437) (Figure 3B). Moreover, the three-, six-, and twelve-month PFS rates were 63.7, 41.5 and 15% in patients with a non-cirrhotic liver, and 53.8, 35.9 and 9% in patients with cirrhosis.

### 3.4. Risk Factors Correlating with Overall Survival in iCCA Patients

As our results indicate a substantial impact of liver cirrhosis on the OS rates of iCCA patients in our study, we further performed univariate and multivariate Cox regression analyses to identify correlating risk factors. Interestingly, the univariate analysis determined liver cirrhosis as a significant risk factor of OS in iCCA patients undergoing gemcitabine-based chemotherapy (HR = 2.3, 95% CI = 1.2–4.2, *p =* 0.017). In addition, the presence of hepatolithiasis could also be described as a significant risk factor (HR = 2.2, 95% CI = 1.2–4.5, *p =* 0.016). However, multivariate analysis revealed that neither liver cirrhosis nor hepatolithiasis serve as independent risk factors for OS in iCCA patients. Furthermore, we identified ECOG performance status 1, positive CA-19/9 at initial diagnosis, irresectability, the presence of distant metastasis, lymph node metastasis, viral hepatitis and diabetes as risk factors of OS (Table 2). Notably, both liver cirrhosis and hepatolithiasis were not identified as risk factors of PFS rates in our study (data not shown).

## 4. Discussion

Studies that address the impact of liver fibrosis in patients with iCCA receiving palliative chemotherapy are missing to date. Therefore, the present study aimed to investigate the role of liver fibrosis in the context of survival in patients undergoing gemcitabine-based chemotherapy as a first-line regimen.

The results of the present study demonstrate that a high liver fibrosis score was associated with reduced OS and PFS, though it was not significant. In the case of cirrhosis, rates were significantly diminished for OS but not for PFS. A plethora of studies analyzed the impact of liver fibrosis or cirrhosis on the survival of patients after surgical resection of an iCCA [15,16,17,23]. For example, a study by Li et al. could identify cirrhosis as an unfavorable prognostic factor in surgically treated iCCA [18]. In contrast, different studies revealed that cirrhosis had no significant impact on prognosis after surgical resection [23,24,25]. Recently, two SEER (Surveillance, Epidemiology, and End Results)-based analyses investigated the impact of fibrosis in large cohorts on survival after surgery and non-surgical therapies. Thereby, Zhang et al. demonstrated that high fibrosis scores were associated with poor clinical outcomes [15]. Correspondingly, Levy et al. showed that advanced liver fibrosis has an increased risk of both overall and cancer-specific mortality [17]. The SEER-based analyses defined fibrosis as a fibrosis score of 5 or 6, referring to the score of Ishak et al., which seems to be comparable with scores of 3 and 4 of the Desmet classification, which is commonly used in our clinical routine and which was used for the present study [19,26]. Due to the available data, they did not investigate the impact of fibrosis in a cohort receiving only chemotherapy. A retrospective single-center study of Jesper et al. identified no significant difference in survival between cirrhotic and non-cirrhotic patients with iCCA receiving chemotherapy (n = 73) [16]. In this study, only cirrhosis, but not fibrosis was examined, and chemotherapy regimens were not specified, making it difficult to draw conclusions on the current first-line standard. This highlights the importance of the data of the current study, where we investigated a well-defined cohort of patients receiving only gemcitabine-based chemotherapy and a histopathological examination was performed by an expert hepatobiliary pathologist. Our results demonstrate that patients with fibrosis should not be prevented from receiving standard-of-care chemotherapy. In the case of cirrhosis, other potential clinically relevant factors such as performance status and expansion of the tumor need to be considered in the decision of chemotherapy regimen and intensity. Thus, a multi-dimensional assessment of cirrhotic patients is mandatory regarding chemotherapy regimens. To correctly interpret the results of the current study, it needs to be considered that we determined liver fibrosis in tissue from resected tumors as well as from biopsies. Thus, it was not always possible to make a delineation with absolute certainty between potential peritumoral fibrosis and fibrosis due to chronic liver disease, although the FIB-4 index corroborates the presence of systemic fibrosis in the present study. It thereby remains to be investigated in further research, whether the potential prognostic relevance derives from the reduced tolerability of chemotherapy due to general liver fibrosis or a potential negative prognostic impact of peritumoral fibrosis. Notably, for other tumor entities such as pancreatic head cancer, peritumoral fibrosis was shown to be associated with worse survival [27]. In CCA patients, cancer-associated fibroblasts (CAF), as well as extracellular matrix proteins, were shown to be associated with tumor growth and reduced survival [28,29,30]. Future prospective trials are warranted investigating the presence and prognostic impact of the tumor microenvironment and cancer progression in iCCA.

We acknowledge the following limitations of our trial. As a single-center study, the sample size was small, especially for the group of cirrhotic patients. Nevertheless, the number of cases of our cohort was within the mean of other comparable studies and was generally limited due to the relatively rare existence of iCCA [18,24]. Given the fact that we analyzed a single-center cohort, our clinical data, including the date of histological confirmation, chemotherapy regimens and follow-up, were comprehensive. Importantly, in contrast to huge population-based datasets, the liver fibrosis score was assessed by an expert gastrointestinal pathologist, strengthening the quality of our data.

In conclusion, this is the first study addressing the prognostic impact of fibrosis and cirrhosis in patients undergoing gemcitabine-based chemotherapy. This study demonstrated that fibrosis has no significant impact on OS and PFS. This shows that these patients should not be prevented from state-of-the-art chemotherapy. However, the intensity of the therapy should be chosen with care in these patients, especially in cases with other clinically relevant factors such as expansion of the tumor and performance status.

## Figures and Tables

**Table 1:** Baseline characteristics of iCCA patients with low and high fibrosis score.

Characteristics	Patients with Low Fibrosis Score (n = 49) No. (%)	Patients with High Fibrosis Score (n = 21) No. (%)	*p-*Value
Sex			0.459
Female	16 (32.7)	5 (23.8)	
Male	33 (67.3)	16 (76.2)	
Age at initial diagnosis			0.4
mean, years (range)	60.7 (25–82)	63.4 (37–79)	
First chemotherapy			0.442
Gem/Cis	40 (81.6)	18 (85.7)	
Gem mono	3 (6.1)	3 (14.3)	
Gem/Ox	4 (8.2)	0 (0)	
Gem/Sor	1 (2)	0 (0)	
Gem/Cis/Dur	1 (2)	0 (0)	
Child–Pugh score			0.186
A (5)	40 (81.6)	14 (66.7)	
A (6)	5 (10.2)	3 (14.3)	
B (7)	3 (6.1)	3 (14.3)	
n.a.	1 (2)	1 (4.8)	
ECOG			0.434
0	38 (77.6)	18 (85.7)	
1	11 (22.4)	3 (14.3)	
2	0 (0)	0 (0)	
CA-19/9 (ng/mL)			0.785
<37	17 (34.7)	8 (38.1)	
≥37	32 (65.3)	13 (61.9)	
Tumor size (cm)			0.174
≤5	20 (40.8)	5 (23.8)	
>5	29 (59.2)	16 (76.2)	
Irresectable			0.617
Yes	32 (65.3)	15 (71.4)	
No	17 (34.7)	6 (28.6)	
Stage			0.394
Locally advanced	18 (36.7)	10 (47.6)	
Metastatic	31 (63.3)	11 (52.4)	
Lymph node metastasis			0.424
No	20 (40.8)	11 (52.4)	
Yes (regional)	14 (28.6)	3 (14.3)	
Yes (distant)	15 (30.6)	7 (33.3)	
8th UICC stage			0.218
Ia	2 (4.1)	0 (0)	
Ib	3 (6.1)	1 (4.8)	
II	6 (12.2)	8 (38.1)	
IIIa	3 (6.1)	1 (4.8)	
IIIb	6 (12.2)	1 (4.8)	
IV	29 (59.2)	10 (47.6)	
FIB-4 score			<0.001
mean (range)	1.9 (0.6–7.4)	4 (1.4–14.9)	
Hepatolithiasis			0.097
Yes	6 (12.2)	6 (28.6)	
No	43 (87.8)	15 (71.4)	
Viral hepatitis			0.026
Yes	7 (14.3)	8 (38.1)	
No	42 (85.7)	13 (61.9)	
Diabetes			0.248
Yes	10 (20.4)	7 (33.3)	
No	39 (79.6)	14 (66.7)	

Abbreviations: CA-19/9 (carbohydrate antigen 19-9), Cis (cisplatin), Dur (durvalumab), ECOG (Eastern Cooperative Oncology Group), FIB-4 (fibrosis-4), Gem (gemcitabine), N.a. (not available), No. (number), Ox (oxaliplatin), Sor (sorafenib), UICC (Union for International Cancer Control).

**Table 2:** Univariate and multivariate Cox regression analysis of overall survival in iCCA patients.

	Univariate Analysis			Multivariate Analysis		
Characteristics	HR 95% CI *p*-Value			HR 95% CI *p*-Value		
Sex						
Female	ref					
Male	0.719	0.413–1.252	0.244			
ECOG						
0	ref					
1	1.416	0.755–2.654	0.278			
CA-19/9 (ng/mL)						
<37	ref					
≥37	1.539	0.886–2.673	0.126			
Tumor size (cm)						
≤5	ref					
>5	0.905	0.529–1.547	0.714			
Irresectable						
No	ref					
Yes	1.211	0.7–2.095	0.494			
Stage						
Locally advanced	ref					
Metastatic	1.205	0.704–2.061	0.496			
Lymph nodes metastasis						
No	ref					
Yes (regional)	1.1	0.561–2.156	0.781			
Yes (distant)	1.24	0.685–2.245	0.477			
FIB-4 score						
≤3.25	ref					
>3.25	1.18	0.649–2.168	0.579			
High fibrosis score						
No	ref					
Yes	0.67	0.382–1.176	0.163			
Liver cirrhosis						
No	ref			ref		
Yes	2.213	1.153–4.248	0.017	1.86	0.886–3.894	0.101
Viral hepatitis						
No	ref					
Yes	1.065	0.561–2.021	0.847			
Hepatolithiasis						
No	ref			ref		
Yes	2.3	1.17–4.52	0.016	0.575	0.267–1.237	0.157
Diabetes						
No	ref					
Yes	1.324	0.733–2.394	0.352			

Abbreviations: CA-19/9 (carbohydrate antigen 19-9), CI (confidence interval), ECOG (Eastern Cooperative Oncology Group), FIB-4 (fibrosis-4), HR (hazard ratio), No. (number).

## Data Availability

All data can be obtained from authors.

## Supplementary Materials

The following are available online at https://www.mdpi.com/article/10.3390/jcm11072057/s1, Table S1: Second- and third-line chemotherapy regimens, Figure S1: Workflow for screening, patient selection and enrollment, Figure S2: Kaplan–Meier curves for overall and progression-free survival in low and high liver fibrosis groups for patients undergoing initial surgical resection, Figure S3: Kaplan–Meier curves for overall and progression-free survival in low and high liver fibrosis groups for unresectable patients at time of diagnosis.
