# Supplementary material for: Impact of Liver Fibrosis on Survival of Patients with Intrahepatic Cholangiocarcinoma Receiving Gemcitabine-Based Chemotherapy
Source: J Clin Med. 2022 Apr 6;11(7):2057. doi: 10.3390/jcm11072057 (PMC8999345; doi:10.3390/jcm11072057)
Supplement: Supplementary file 1 [file jcm-11-02057-s001.zip › jcm-1654726-supplementary.pdf]

Supplementary Materials

Supplementary Table S1. Second- and third-line chemotherapy

Chemotherapy	**Patients with second-line chemotherapy (n=45)** No. (%)	**Patients with third-line chemotherapy (n=16)** No. (%)
Gemcitabine/Cisplatin	2 (4.4)	4 (25)
Gemcitabine mono	8 (17.8)	-
Gemcitabine/Oxaliplatin	1 (2.2)	1 (6.3)
Capecitabine	3 (6.7)	-
Folfox	4 (8.9)	1 (6.3)
Folfiri	17 (37.8)	2 (12.5)
5FU	1 (2.2)	2 (12.5)
BAY18239	1 (2.2)	-
Capecitabine/Oxaliplatin	1 (2.2)	-
Capecitabine/Irinotecan	2 (4.4)	-
Folfirinox	1 (2.2)	-
Anti-PDL1-TGF	2 (4.4)	-
BTC047-study	2 (4.4)	-
Nivolumab	-	1 (6.3)
Irinotecan	-	1 (6.3)
Ivosidenib	-	1 (6.3)
Denosumab	-	1 (6.3)
Capecitabine/Docetaxel	-	1 (6.3)
Gemcitabine/Cisplatin/nabPaclitaxel	-	1 (6.3)

Abbreviations: 5-FU (Fluorouracil), Folfox (Fluorouracil / Folinic acid / Oxaliplatin), Folfiri (Fluorouracil / Folinic acid / Irinotecan)

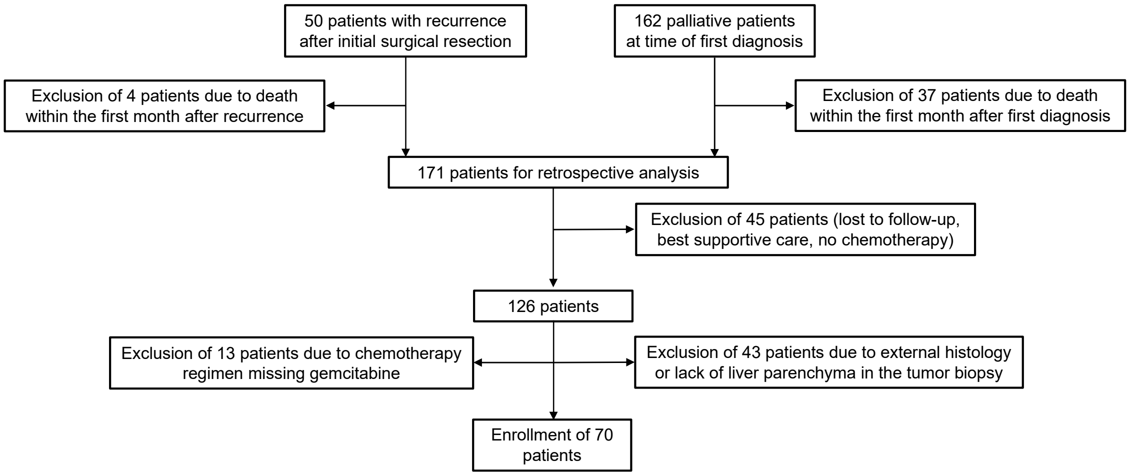

Supplementary Figure S1. Workflow for screening, patient selection and enrollment.

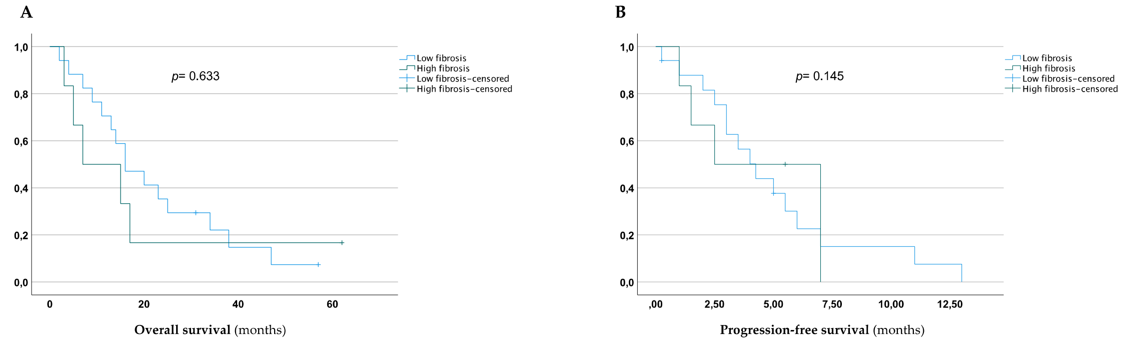

high liver fibrosis group for patients undergoing initial surgical resection. **A/B** Overall survival (**A**) and progression-free survival (**B**) assessed for low and high liver fibrosis score. Date of last follow-up was treated as censored observation.

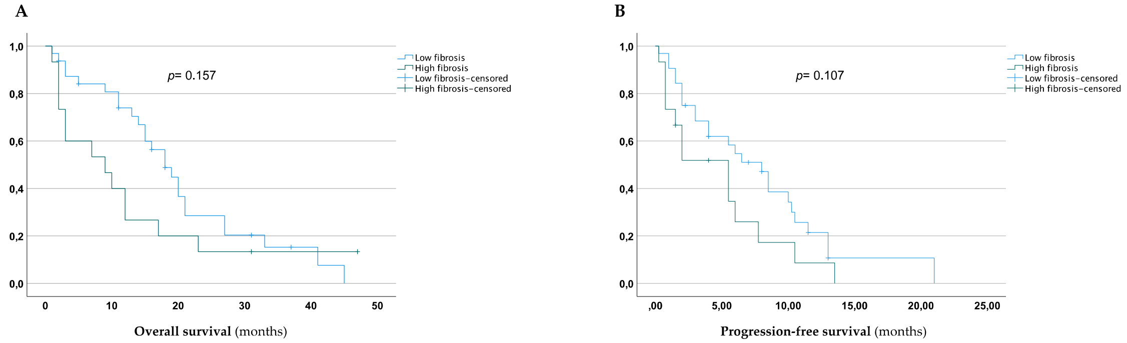

Supplementary Figure S3. Kaplan Meier curves for overall- and progression-free survival in low and high liver fibrosis group for unresectable patients at time of diagnosis. **A/B** Overall survival (**A**) and progression-free survival (**B**) assessed for low and high liver fibrosis score. Date of last follow-up was treated as censored observation.
